# Design of a Cavity for the High-Power Radio-Frequency Quadrupole Coupler Test for the ANTHEM Project

**DOI:** 10.3390/s24134165

**Published:** 2024-06-26

**Authors:** Andrea Passarelli, Maria Rosaria Masullo, Carlo Baltador, Francesco Grespan, Antonio Palmieri, Andrea Pisent, Paolo Mereu, Carlo Mingioni, Marco Nenni, Edoardo Nicoletti

**Affiliations:** 1 National Institute for Nuclear Physics, Naples Unit, Via Cintia, 80126 Naples, Italy; masullo@na.infn.it; 2 National Institute for Nuclear Physics, National Laboratories of Legnaro, Viale dell’Università, 2, 35020 Legnaro, Italy; 3 National Institute for Nuclear Physics, Turin Unit, Via Pietro Giuria, 1, 10125 Turin, Italycarlo.mingioni@to.infn.it (C.M.);

**Keywords:** radio-frequency quadrupole, linear accelerator, coupler, high-power test, cavity design, boron neutron capture therapy

## Abstract

The ANTHEM (Advanced Technologies for Human-centered Medicine) Radio-Frequency Quadrupole (RFQ) will employ eight coaxial power couplers, which will be magnetically coupled to the device through a loop antenna. The coupler design can support up to 140 kW in continuous wave operation. This paper presents the design of the cavity used for high-power testing, with the primary objectives of both optimizing the coupling between the couplers and ensuring operations at the designated operating frequency. Furthermore, the paper encompasses thermal and structural assessments conducted through numerical simulations.

## 1. Introduction

The ANTHEM (Advanced Technologies for Human-centered Medicine) research project will establish a Research and Clinical Center in Caserta, Italy, for the study and application of Boron Neutron Capture Therapy (BNCT), an innovative radiotherapy approach designed to tackle the complexities of infiltrating, radioresistant, or metastatic tumors [1]. This achievement will be made possible through the refurbishment of a Radio-Frequency Quadrupole (RFQ) accelerator, originally designed at the National Laboratories of Legnaro of INFN (National Institute for Nuclear Physics) [2,3,4]. The RFQ operates with eight coaxial power couplers, magnetically coupled to the cavity via a loop antenna.

The high-power testing (conditioning) of the couplers before installation on RFQ is crucial to verify their design and functionality and minimize the condition of their damage. Conditioning involves gradually exposing the couplers to higher peak and average powers, even surpassing their nominal operating values. This process ensures the coupler performance before integration into an accelerator, with particular attention to the dielectric material used as an insulator (i.e., alumina), and may need to be repeated periodically during operation. Conditioning must be conducted safely and under control in dedicated test structures [5]. Upon the initial exposure to RF power, there are significant increases in pressure within the test structure vacuum, accompanied by the detection of electron signals. These vacuum bursts typically occur at specific power levels that correspond to the exponential multiplication of secondary electrons emitted from the walls in resonance with the RF field, known as multipactor resonances [6]. The interaction of the avalanche of electrons with the coupler surfaces causes the desorption of adsorbed gases, which leads to a decrease in multipactor intensity, likely due to a reduction in the Secondary Electron Emission coefficient. This surface process must continue until vacuum bursts and electron signals cease to occur at any operating power level of the coupler, so suppressing the multipacting discharge [7].

Additionally, it may be convenient to conduct coupler tests in pairs, as the RF windows isolate the upstream and downstream waveguides from the coupler vacuum.

In the realm of the literature, advancements have been made in the development of test infrastructure. An example is the creation of a test stand accompanied by an LLRF (Low-Level Radio Frequency) control system featuring multiple conditioning modes and rapid interlocks. This setup was specifically designed for a coupler operating at 162.5 MHz. To facilitate non-contact conditioning, a multi-purpose test cavity was designed and optimized [8]. Another instance involves a room-temperature stand for a Radio Frequency (RF) test at 1.3 GHz, comprising a rectangular waveguide coupled with two power couplers along with the necessary instrumentation for controls and data acquisition. The studied power couplers are intended for 1 MW pulsed power applications, although typically operated at no more than 1.5 kW average power. Notably, 20 sets of prototypes underwent testing on this room-temperature RF test stand, demonstrating transfer capabilities of up to 14 kW in the traveling wave and 7 kW in the standing wave. Additionally, an automated RF test bench was installed to enhance efficiency [9]. Furthermore, a high-power test was conducted using standing waves reaching up to 20 kW at 325 MHz. Despite encountering multipacting effects across a broad RF power spectrum, these were effectively mitigated through RF conditioning during operation, particularly at 3 kW power levels. Moreover, temperature assessments revealed a modest rise in component temperatures of the power coupler’s RF window, remaining within a range of 30 °C [10]. Fermilab has designed and tested two prototype couplers for the SSR-type cavities used in the Project X Injector Experiment (PXIE). This experiment includes a cryomodule containing eight 325 MHz single-spoke superconducting cavities. Each cavity requires approximately 2 kW of continuous wave (CW) RF power to operate with a beam current of 1 mA. The couplers were tested for power transmission at approximately 7.8 kW CW for a duration of 7 continuous hours [11]. Additional examples in the literature illustrate two distinct types of test setups, employing either a cavity [12,13,14,15] or a waveguide [16,17,18,19] for conducting tests.

In this paper, we conducted a comprehensive design, both electromagnetically and thermomechanically, of the test device used for the ANTHEM RFQ couplers, a bridge cavity, that is a resonant structure tuned to the same operating frequency as the couplers (352.2 MHz). The cavity features two ports to facilitate coupler conditioning, one connected to the RF source and the other connected to a water-cooled load. A similar configuration has been previously employed for comparable couplers, and HFSS simulations have indicated that approximately 30% of the input power dissipates on the cavity walls, as documented in [20]. The goal of this paper is to achieve a cavity design that enhances coupling between the couplers, thereby minimizing power dissipation.

## 2. Basic Elements of the Design

**Figure 1:**
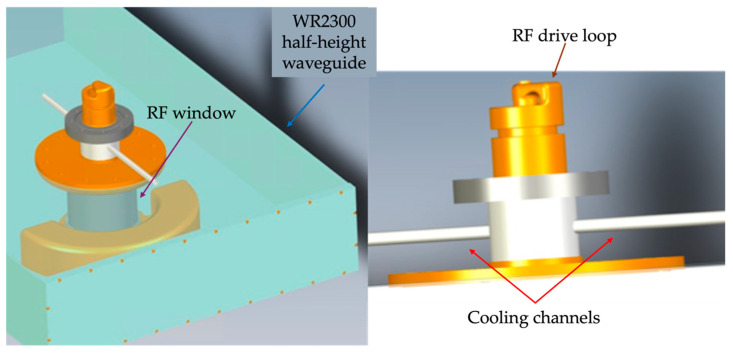
Three-dimensional render of the coupler.

The RF coupler design, based on the one detailed in [21], comprises the drive loop, the coaxial transmission line—completed with its corresponding cooling channels—and the coaxial alumina window (as shown in Figure 1).

**Figure 2:**
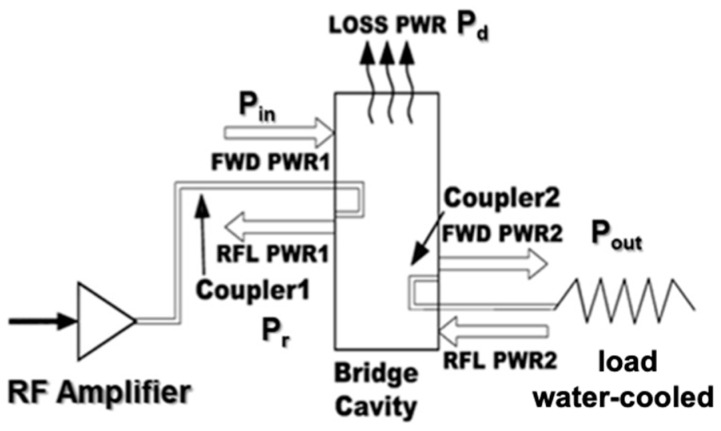
Functional scheme of the coupler tests.

Figure 2 illustrates the functional scheme of the coupler high-power tests. A resonant structure, which operates at half the wavelength of the operating frequency of 352.2 MHz, has been chosen. The cavity includes two ports, one linked to the source and the other connected to a water-cooled load.

The design of the bridge cavity structure has been carefully considered both to maximize the power transfer between couplers and to resonate at the RFQ operational frequency. For a generic cavity with two couplers, the incident power, for each coupler, $P_{in}$ is defined as

(1) $$P_{in}=P_{ref}+P_{cav}+P_{ext}={\left|\Gamma \right|}^{2}P_{in}+P_{cav}+\beta P_{cav},$$

where $P_{ref}$ is the reflected power, $P_{cav}$ is the dissipated power inside the cavity, and $P_{ext}$ is the power on the coupler. *β* is the coupling factor between the input coupler and the cavity and Γ is the reflection coefficient at the input port. The ratio between the power in the cavity and the input is

(2) $$\frac{P_{cav}}{P_{in}}=\frac{1-{\left|\Gamma \right|}^{2}}{1+\beta }.$$

Considering the same coupling factors for the two couplers $\beta_{1}=\beta_{2}$, the reflection coefficient is [22]

(3) $$\Gamma =\frac{1}{1+2\beta }.$$

In these conditions, we derive the ratio between the power dissipated inside the cavity and the input power as

(4) $$\frac{P_{cav}}{P_{in}}=\frac{4\beta }{{\left(1+2\beta \right)}^{2}}.$$

The external power, under the hypothesis that the magnetic *H* field is constant in the loop area, is approximately equal to [22]

(5) $$P_{ext}=\beta P_{cav}=\frac{{\left(\mu_{0}\omega H_{0}S\right)}^{2}}{2Z_{coup}},$$

with *μ*_0_ as the magnetic permeability in a vacuum, ω=2πf, $H_{0}$ as the magnetic field strength at the coupler loop position in the bridge cavity, *S* = 2.4 cm^2^ as the effective area of the coupler loop, and $Z_{coup}$ = 40 Ω as the coupler port impedance. The coupler loop position (penetration) in the bridge cavity is the same as it will be in RFQ. The power dissipated within the cavity is

(6) $$P_{cav}=\frac{R_{S}}{2}\iint {\left|{\hat{i}}_{n}\times H\right|}^{2}\;dS,$$

where $R_{S}=\sqrt{\mu_{0}\omega /2\sigma }$ is the RF surface resistance of copper at the operating frequency and *H* is the magnetic field integrated on the cavity surface.

### 2.1. Transmission Line Equivalent Circuit of the Bridge Cavity

**Figure 3:**
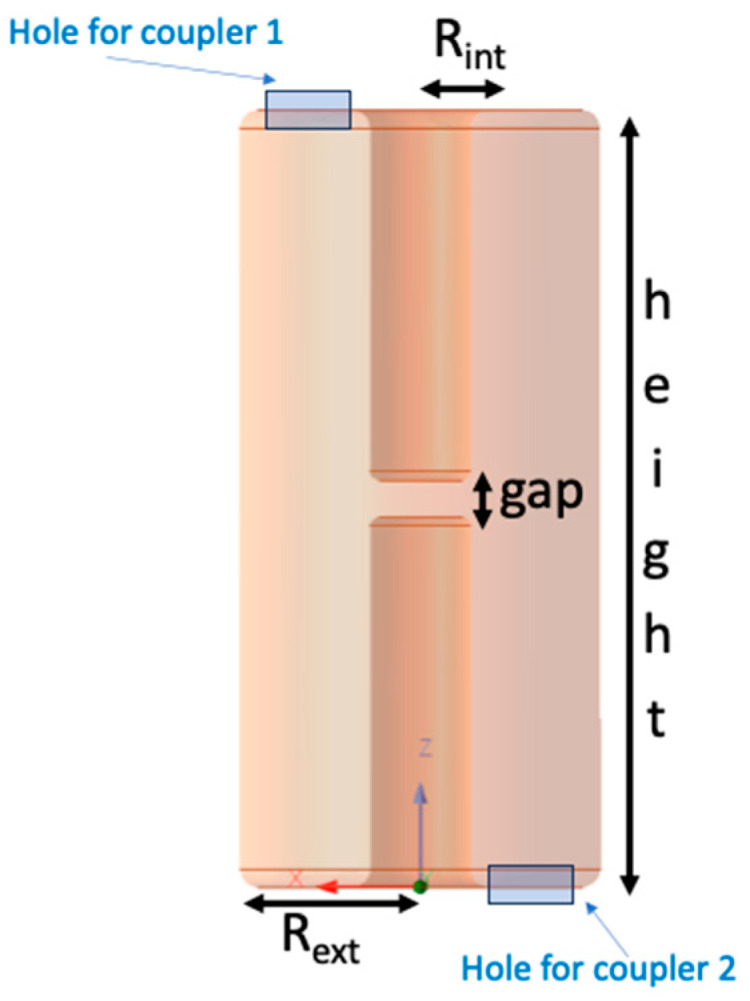
Design parameters of the bridge cavity.

The structural design of the cavity was based on the specific area requirements of the couplers. The primary objective was to maximize the magnetic field at the coupler loop height. We chose a coaxial cavity with a centrally divided post, as illustrated in Figure 3. Initially considering a λ/4 configuration, we faced size limitations preventing the insertion of loops. Consequently, we shifted to a λ/2 resonator, as its dimensions allow for the insertion of two couplers on the flat sections.

**Figure 4:**
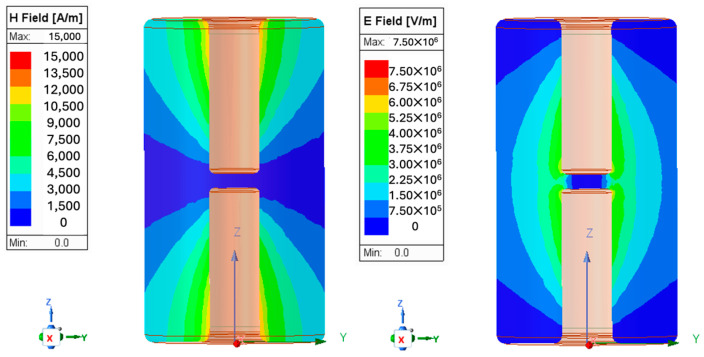
Magnetic and electric field distributions for the resonant mode at 352.2 MHz.

To simplify construction and assembly, taking advantage of the electric and magnetic field distributions inside the cavity, the central post was divided into two separate sections. In Figure 4, the electric *E* and magnetic *H* field distributions are shown, corresponding to the actual dissipated power into the bridge cavity.

**Figure 5:**
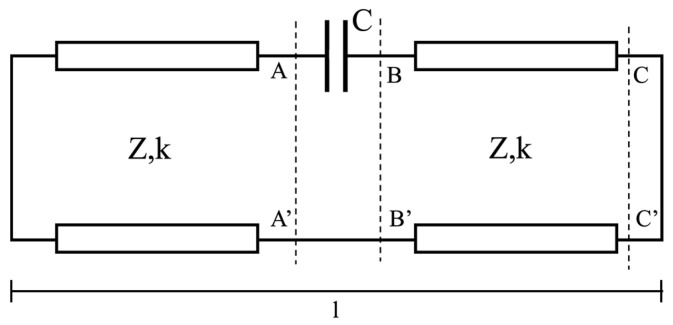
Transmission line equivalent circuit of the bridge cavity.

To first estimate the geometrical parameters of the cavity, we utilized an equivalent transmission line (TL) circuit (see Figure 5).

The gap between the two pieces of the post is represented by the capacitance *C* and the two transmission lines represent the coaxial cables formed by the cylinders inside the structure, where the length *l* corresponds to the total height of the structure.

The general formula we use for impedance transfer is [23]

(7) $$Z=Z_{0}\frac{Z_{L}+jZ_{0}tan(kd)}{Z_{0}+jZ_{L}tan(kd)},$$

where $Z_{L}$ is the load impedance, $Z_{0}$ is the transmission line impedance of the length d, and k is the wave number. In the particular case of Figure 5, we can express the short circuit transfer ($Z_{L}=0$) on the first transmission line ($Z_{0}=Z_{coax}$) as

(8) $$Z_{{AA}^{\prime }}=jZ_{coax}tan(kl/2),$$

where $Z_{coax}$ is the characteristic impedance of a coaxial line:

(9) $$Z_{coax}=\frac{\zeta_{0}}{2\pi }\ln\frac{R_{ext}}{R_{int}},$$

where $\zeta_{0}=\sqrt{\frac{\mu_{0}}{\varepsilon_{0}}}=376.73\;\Omega$ is the characteristic impedance in the vacuum and $R_{ext}$ and $R_{int}$ are the external and internal cavity radii, respectively. The impedance $Z_{{BB}^{\prime }}$ is the series of $Z_{{AA}^{\prime }}$ and the capacitance $C=\varepsilon_{0}\frac{\pi R_{int}^{2}}{gap}$ of the parallel-plate resonator:

(10) $$Z_{{BB}^{\prime }}=Z_{{AA}^{\prime }}+\frac{1}{j\omega C}.$$

Transporting the impedance $Z_{{BB}^{\prime }}$ onto the second transmission line results in the following impedance:

(11) $$Z_{{CC}^{\prime }}=Z_{0}\frac{Z_{{BB}^{\prime }}+jZ_{0}tan(kl/2)}{Z_{0}+jZ_{{BB}^{\prime }}tan(kl/2)}.$$

The resonance condition occurs when $Z_{{CC}^{\prime }}=0$ and is used to estimate the total length of the cavity l.

### 2.2. Coaxial Resonator Approximation

**Figure 6:**
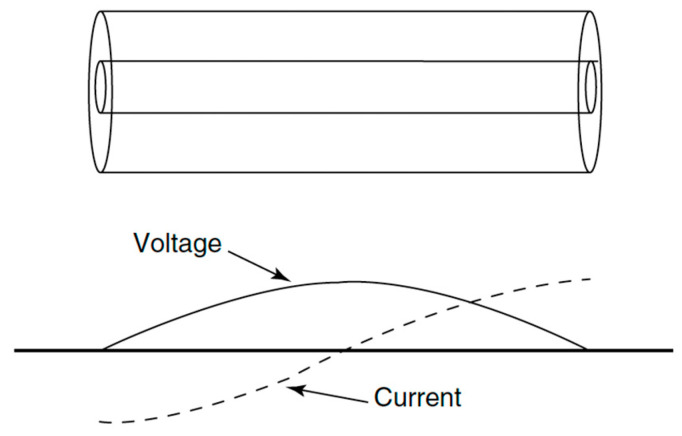
Coaxial resonator with voltage and current standing half-wave.

For an initial assessment of the coupling coefficient β, we can approximate our cavity with a half-wavelength coaxial resonator (see Figure 6).

The electromagnetic stored energy U is

(12) $$U=\frac{\mu_{0}l\;I_{0}^{2}\ln\left(\frac{R_{ext}}{R_{int}}\right)}{2\pi },$$

where $I_{0}\;$ is the current inside the cavity. The quality factor $Q_{0}$, including the losses on the end walls, is

(13) $$Q_{0}=\frac{\pi }{R_{S}}\sqrt{\frac{\mu_{0}}{\varepsilon_{0}}}\frac{\ln\left(\frac{R_{ext}}{R_{int}}\right)}{\left[l\left(\frac{1}{R_{int}}+\frac{1}{R_{ext}}\right)+4\ln\left(\frac{R_{ext}}{R_{int}}\right)\right]}.$$

Formulae (12) and (13) are taken from Equations (1.30) and (1.31) in [22], respectively, considering the dissipated power inside a cavity as

(14) $$P_{cav}=2\pi f\frac{U}{Q_{0}}.$$

By substituting the result of this equation into Equation (5), we obtain the coupling factor for a half-wave coaxial resonator:

(15) $$\beta =\frac{2\pi f\mu_{0}\zeta_{0}S^{2}}{lR_{S}Z_{coup}r^{2}}\frac{\cos^{2}\left(\frac{\pi z}{l}\right)}{\left[l\left(\frac{1}{R_{int}}+\frac{1}{R_{ext}}\right)+4\ln\left(\frac{R_{ext}}{R_{int}}\right)\right]}.$$

where r and z are the radius and height, respectively, relative to the reference plane of the flat wall on which the coupler is positioned.

## 3. Geometrical Parameters

To facilitate the insertion of the couplers on the two flat cavity plates, the external cavity radius $R_{ext}$ is set at 120 mm. To enhance the Q-factor and minimize losses within the structure, the ratio between the external and internal radii was set to 3.6, following established principles in microwave engineering [24]. Consequently, the internal radius $R_{int}$ resulted at 33.33 mm with an adequate coupler insertion space of approximately 86.67 mm.

**Figure 7:**
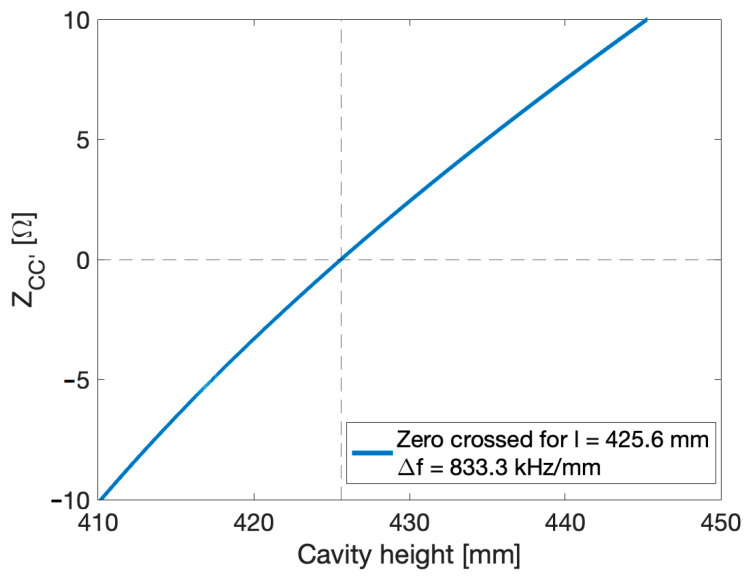
The impedance $Z_{{CC}^{\prime }}$ as a function of cavity height l.

From the transmission line equivalent circuit, we estimated a first approximation of the total height of the cavity. Figure 7 illustrates the assessment of the impedance $Z_{{CC}^{\prime }}$, which is equal to zero (as per Equation (11)) for a total cavity length l = 425.6 mm, with a central gap g = 20.0 mm.

A study on the variation of the cavity total length, with the transmission line equivalent circuit, gives a frequency shift of 833.3 kHz per millimeter.

**Figure 8:**
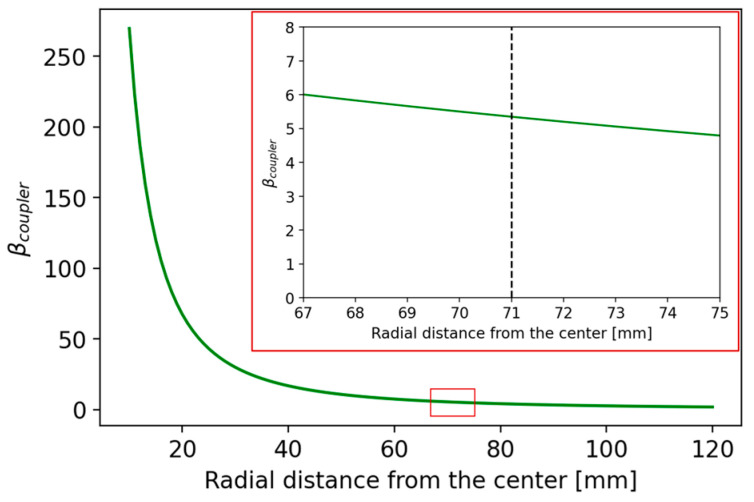
Coaxial resonator approximation: coupling factor as a function of the radial distance from the center r; magnification is framed in red.

Considering the previous cavity model, which approximates the coaxial resonator, the coupling factor was evaluated using Equation (15) for a coupler insertion depth of z = 20 mm, which matches the real depth inside the RFQ. In Figure 8, the coupling factor β is shown as a function of the radial distance from the cavity center r evaluated with the coaxial resonator approximation.

For a value of r = 71 mm, representing a trade-off distance to facilitate coupler insertion and maximize the coupling factor, the model yields a coupling factor of $\beta_{coupler}$ = 5.35 and a ratio of dissipated power to input power equal to 15.6%.

The TL circuit approach and the coaxial resonator approximation are useful to initially size the bridge cavity. The final dimensions of the device were derived utilizing an electromagnetic CAD simulator, HFSS [25] simulations in our case, assuring that our cavity design would efficiently resonate at the desired frequency while accommodating the essential components.

To mitigate discharges in the region characterized by the highest electric field strength, we have chosen to smooth out the sharp surfaces at the two extreme edges of the internal posts. Employing a 5 mm radius for this purpose is strategic, given that these areas are located in a less critical magnetic field region. Concurrently, a curvature radius of 10 mm has been applied to both the corner between the post and the plates, as well as the intersections of the plates with the side walls. This decision stems from the fact that the magnetic field reaches its maximum intensity in these specific zones. Hence, the adoption of a larger surface area facilitates more effective power dissipation. Moreover, the smoothing out of sharp surfaces enhances the ease of construction for the final structure.

**Figure 9:**
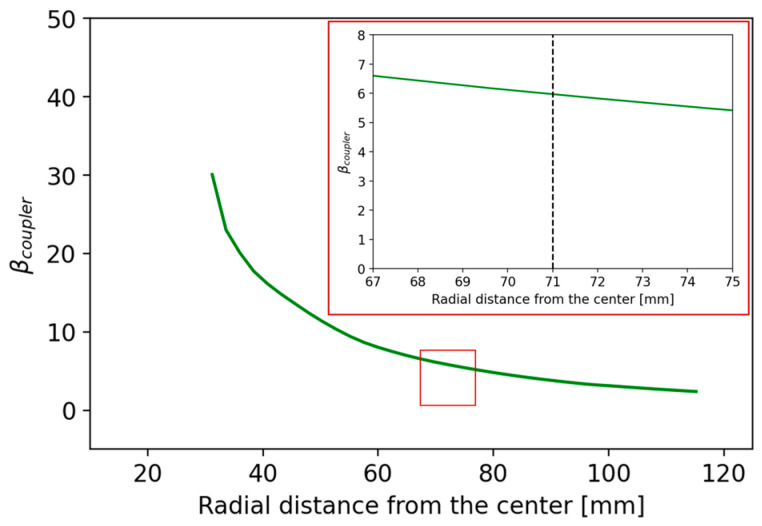
HFSS simulations: coupling factor as a function of the radial distance from the center r; magnification is framed in red.

From the HFSS simulations, using a normal deviation of 10 degrees for the surface approximation of the mesh and applying a curvilinear mesh with approximately 75,000 tetrahedra and considering finite conductivity of copper as boundary conditions, the final geometry of the cavity has a total height of 429.8 mm and a central gap between the two cavity posts of 20.0 mm. With this configuration and the coupler positioned at a radial distance from the center (r = 71 mm) and a height of z = 20 mm, we obtain a ratio between the input power and the trapped power in the cavity of approximately 14.3% and a coupling coefficient *β* on the order of 5.97. In Figure 8, the coupling factor *β* is shown as a function of the radial distance from the center r evaluated with the HFSS simulations. The trend of the graph in Figure 9 and its similarity to that in Figure 8 confirms that the equivalent circuit for transmission lines (TLs) functions as a good initial approximation for parameterizing the cavity. This can be further evaluated and optimized with more precision through electromagnetic simulations.

### 3.1. Mechanical Tolerances Using HFSS

**Figure 10:**
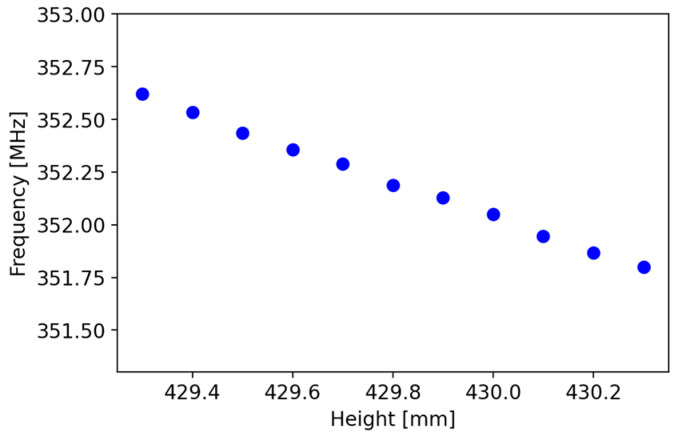
Variation in resonating operating frequency for different values of cavity height.

The analysis of mechanical tolerances allows us to see how changes in geometric parameters impact the cavity resonance frequency. Specifically, the variations in the “height” parameter have a significant impact, with a frequency shift of 820 kHz per millimeter (see Figure 10), in agreement with previous calculations with coaxial resonator approximation.

The “gap” parameter, on the other hand, exhibits an influence of 170 kHz per millimeter. In fixing the radius ratio at 3.6, changes in both internal and external radii have minimal influence on frequency compared to all other parameters.

Regarding the smoothing of sharp surfaces, we examined how the curvature radius affects frequency changes. The most significant impact occurs at the junction between the central post and the plate, causing a 200 kHz change per millimeter. Smoothing the tips of the two posts has a slightly lower effect, with a 180 kHz change per millimeter. The least effect is seen when smoothing the outer edges of the plates, resulting in a 55 kHz change per millimeter. These results underline the importance of precise control over the cavity’s height to achieve the desired resonance frequency since no tuner is foreseen for this application.

The results both for the transmission line circuit and the HFSS simulations, which are the ones used for the final project, are reported in Table 1.

The results both for the coaxial resonator approximation and the HFSS simulations are reported in Table 2.

### 3.2. Power Distribution

For a thermal study of structure, we assessed power dissipation within the cavity by resorting to HFSS simulations. Specifically, considering an input power of 140 kW, we observed a total dissipation of 30 kW. Simulations show that this power dissipation was distributed across various components: 9.25 kW on each internal cylindrical post, 3.15 kW on each external plate, and 5.25 kW on the outer section of the cylinder. These findings reveal that the bulk of the power dissipation occurs on the surface of the internal cylinder.

**Figure 11:**
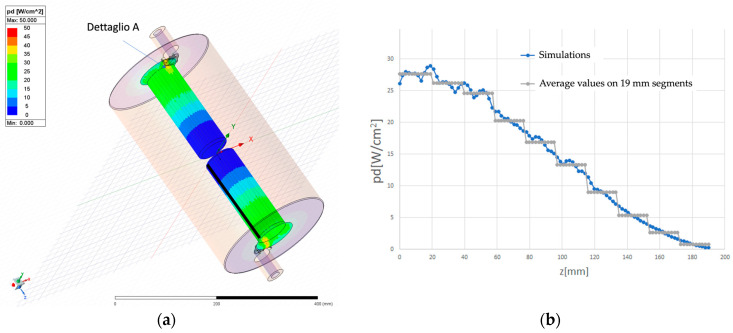
(**a**) Power density inside the internal posts; (**b**) longitudinal power distribution along the black line in (**a**) (blue dots). The black line is divided into 10 segments, each 19 mm long, with the average power density assigned to each segment (gray line).

Figure 11a displays the power density distribution on the internal posts.

**Figure 12:**
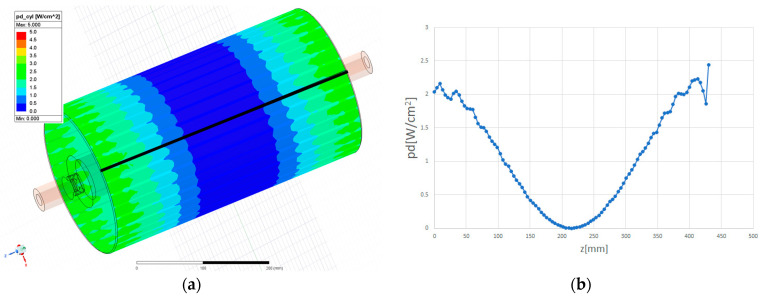
(**a**) Power density distribution on the outer cylinder; (**b**) longitudinal power distribution along the black line in (**a**).

In Figure 11b, the longitudinal distribution of power density, *p*(*x*) [W/cm^2^], along the black line in Figure 11a is depicted. This distribution can be approximated by the function

(16) $$p\left(x\right)=p\left(0\right)\cos^{2}\frac{\pi }{2L}x,$$

where L = 190 mm, the length of the black line, and p(0) = 28.5 W/cm^2^. Figure 12a displays the power density distribution on the outer cylinder.

**Figure 13:**
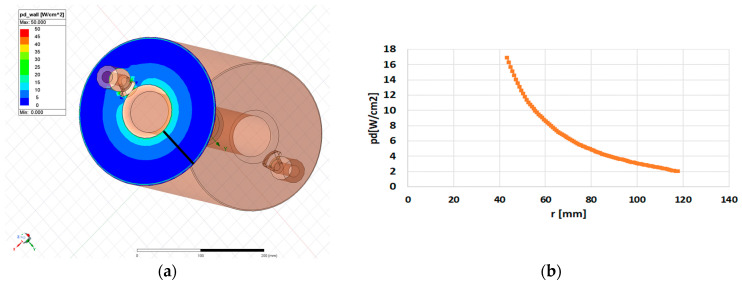
(**a**) Power density distribution on one of the two flat walls [W/cm^2^]. The other one is identical; (**b**) longitudinal power distribution along the black line in (**a**).

In Figure 12b, the longitudinal distribution of power density [W/cm^2^] along the black line in Figure 12a is depicted. This distribution can be approximated by the function

(17) $$p\left(x\right)=p\left(0\right)\cos^{2}\frac{\pi }{2L_{2}}x,$$

where $L_{2}$ = 215 mm, the length of the black line, and p(0) = 2.03 W/cm^2^. Figure 13a displays the power density distribution on one of the two flat walls.

In Figure 13b, the longitudinal distribution of power density [W/cm^2^] along the black line in Figure 13a is depicted. This distribution can be approximated by the function

(18) $$p\left(r\right)=\frac{A}{r^{2}},$$

where A = 31,734 (W/cm^2^) mm^2^ is a fitting parameter.

**Figure 14:**
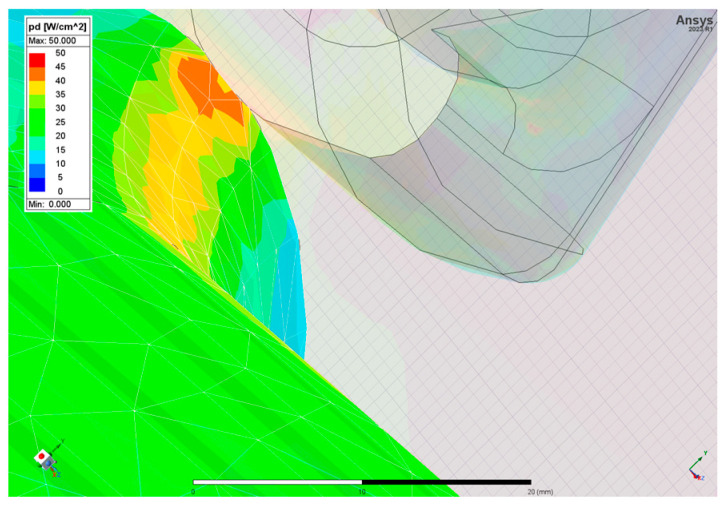
Power density inside the bridge cavity at the juncture where the coupler interfaces with the plates.

Figure 14 displays the power density distribution along the most critical structural element, highlighting a maximum concentration of 32 W/cm^2^ at the juncture where the coupler interfaces with the plates.

These simulations underscore the need for thermal assessments that incorporate cooling circuitry, as it is essential to maintain the structure temperature within acceptable limits and prevent potential overheating issues, which would deform the cavity with consequent deterioration in performance and outgassing problems, leading to the undesirable effect of multipacting and breakdown.

## 4. Mechanical Design

**Figure 15:**
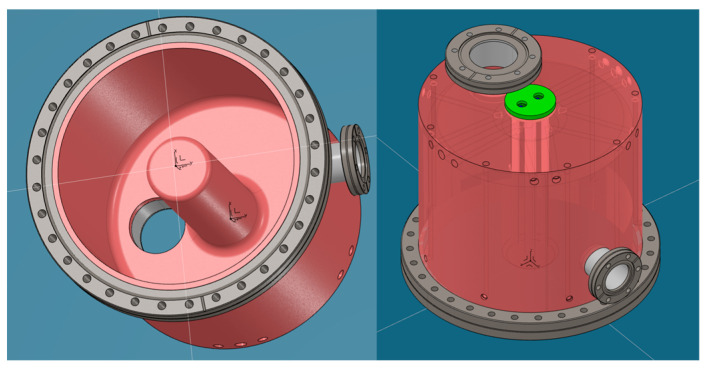
View of the internal (**left**) and complete structure (**right**). The copper structure is in pink. The CF flanges and their respective stainless steel collars are in gray. The end cap for the coaxial cooling channels, made of stainless steel, is in green.

**Figure 16:**
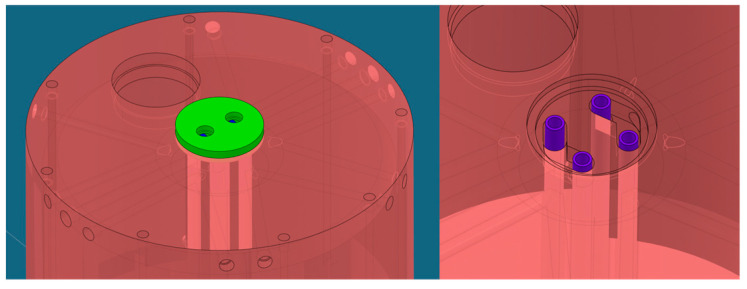
Cooling geometry of the central post: detailed closure cap (**left**) and detailed internal tubes (**right**). The inner tubes of the coaxial cooling channels, made of stainless steel, are shown in purple.

**Figure 17:**
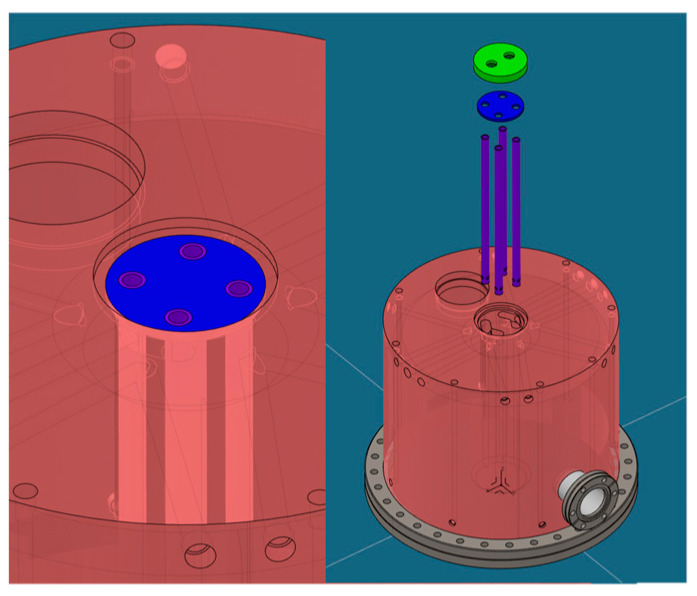
Central coaxial: detailed separator partition (**left**) and exploded view (**right**). The separator for the inlet/outlet of the coaxial cooling channels, made of stainless steel, is highlighted in blue.

A preliminary study was conducted to explore the use of various materials, such as copper and stainless steel AISI 304L, and different cooling geometries. The temperature distribution in stainless steel was found to be over three times that in copper for a cavity with the same configuration of cooling channels. Consequently, copper was chosen as the primary material to enhance heat dissipation within the structure. Considering that significant power is dissipated in the central part of the structure, as shown from previous studies, four water-cooled cooling tubes were placed inside the central post (see Figure 15, Figure 16 and Figure 17): this creates four coaxial distributions where water supply is on each circular crown and water return is inside the tubes.

**Figure 18:**
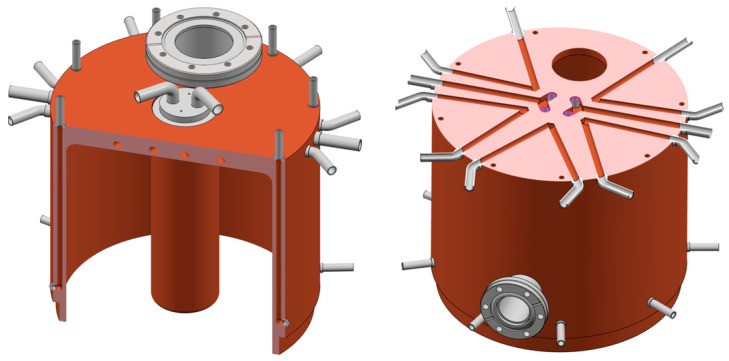
Complete cooling design: detailed view of longitudinal channels (**left**) and V-shaped channels (**right**).

Figure 18 illustrates the complete design of all cooling tubes, which includes longitudinal channels along the cavity walls and V-shaped channels for plate cooling.

The cavity will be manufactured from a bulk copper cylinder. All the tubes for cooling, the flange for the vacuum and coupler connection, and the cap that closes the coaxial and creates the cooling loop will be made of SS AISI 304L and will be brazed to the cavity in a vacuum oven.

## 5. Thermal Design

The cavity is cooled with water at 20 °C. The distribution of the channel is designed to maximize the temperature uniformity. The high-water speed in the channel increases the coefficient of convection *h* but also increases the pressure loss *r*. So, mass flow and channels’ diameter *D* were chosen after an iterative procedure, balancing both parameters. *h* was estimated using Dittus–Boelter correlation since that channel internal surface is smooth and *L*/*D* > 10, where *L* is the channel length and *D* is the inner diameter

(19) h=k Nu/D,

(20) $$Nu=0.023\;Re^{0.8}{Pr}^{0.4},$$

(21) $$Re=\frac{v\;D\;\rho \;}{\mu },$$

(22) $$Pr=\frac{c_{p}\;\mu \;}{k},$$

where *k* is the water thermal conductivity, *c_p_* the isobaric specific heat, *ρ* the density, *µ* the dynamic viscosity, *v* is the averaged speed in the channel section, *Nu* is the Nusselt number, *Re* is the Reynolds number, and *Pr* is the Prandtl number.

For the evaluation of water behavior in a single coaxial channel of the central post, the hydraulic diameter was calculated and used in the correlation. The internal tube of the coaxial is considered adiabatic, so only the water in the circular crown removes heat. In this case,

(23) $$D_{hyd}=D_{ext}-D_{int},$$

where *D_ext_* and *D_int_* are the outer and inner diameters of the circular crown.

The pressure loss *r* and friction factor *f* were evaluated using approximated correlations, valid for water turbulent flow in linear channels.

(24) $$r=\frac{1}{2}\rho \;f\frac{v^{2}}{D},$$

(25) $$f=0.316\;Re^{-0.25}.$$

Table 3 summarizes the parameters evaluated for all the types of channels. It can be seen that high convective coefficients are used where the thermal load is higher, at the price of a high-pressure loss. The evaluated *h* coefficients are then used in FEM (Finite Element Method) simulations to verify and validate the design.

### Thermostructural Evaluations

FEM thermostructural simulations were conducted using Ansys Workbench [25]. The thermal simulations used the evaluated *h* coefficients above. Thermal–structural simulations were made with a mesh of 600,000 elements and 960,000 nodes with first-order interpolation. The Element Quality was evaluated with average µ = 0.78 and standard deviation σ = 0.11. In the thermal simulation, all the surfaces without a thermal load, from RF power or from convection, were set as adiabatic.

**Figure 19:**
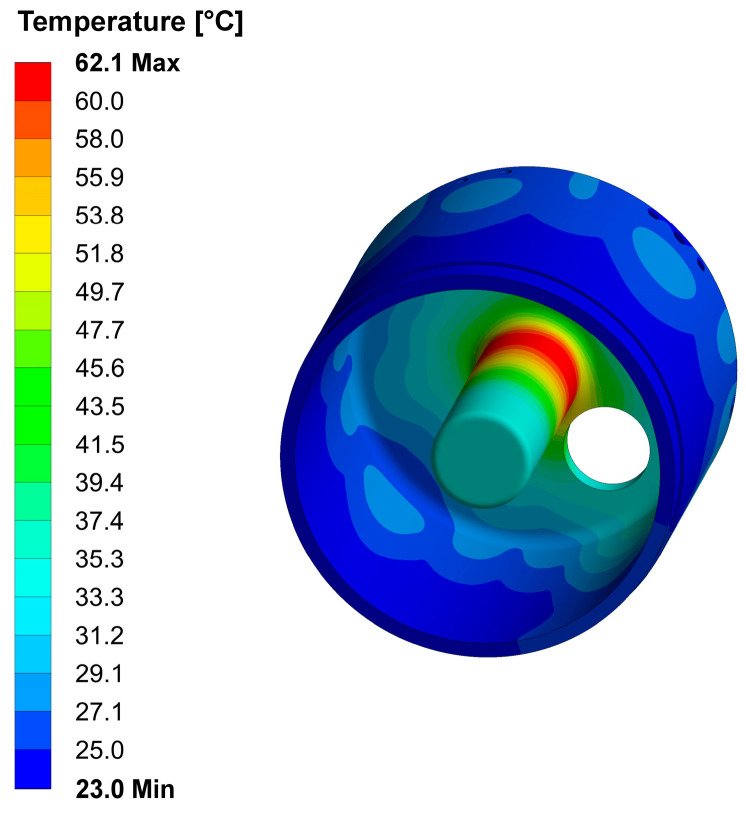
Temperature distribution. The unit of measurement in the color bar is in °C.

The results indicate that the maximum reached temperature is around 62 °C (see Figure 19). This underscores the importance of the cooling circuits.

The structural simulation used the temperature map as input, with a fixed constraint in the lower part of the cavity, and produced the stress and deformation distributions.

**Figure 20:**
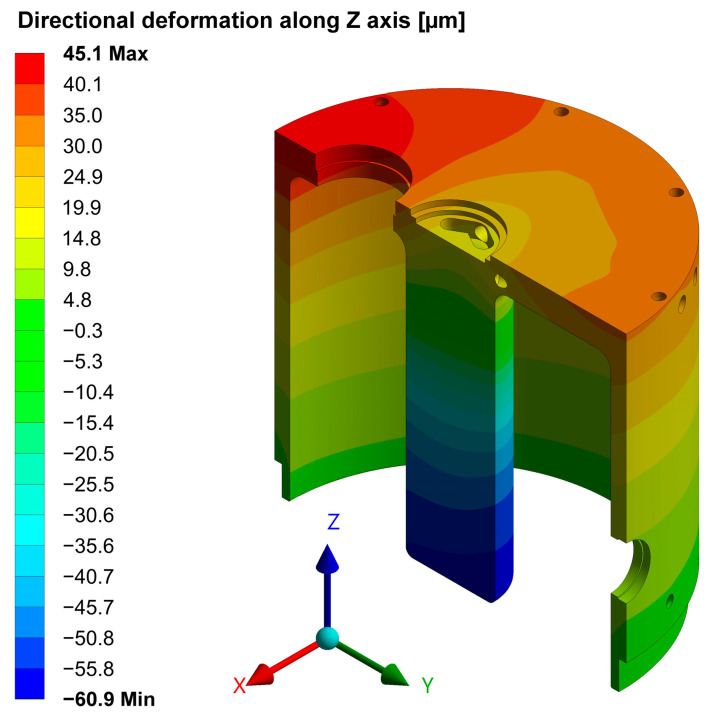
Simulated deformation relative to $T_{0}=20\;{}^{\circ}C$, with respect to the axis of the cavity. The unit of measurement in the color bar is in µm.

Figure 20 illustrates the simulated deformation relative to the operating temperature $T_{0}=20\;{}^{\circ}C$, evaluated with respect to *z*, the axis of the cavity. The structure experiences a maximum absolute deformation of 60 µm, which has minimal impact on its resonance frequency.

## 6. Conclusions

The design of a bridge cavity for high-power coupler testing has been proposed through a detailed exploration of its electromagnetic behavior and a thermostructural evaluation. Starting from the theoretical and numerical analyses of a simplified and then detailed structure, the geometric parameters of the cavity were assessed to achieve the desired resonance frequency of 352.2 MHz. HFSS simulations were conducted to identify geometric tolerances and the distribution of power dissipated within the geometry, considering an input power of 140 kW. The design work on the cavity parameters resulted in a percentage of power dissipated relative to the input power of approximately 14.3%, with a coupling factor *β* of 5.97. Thermostructural analyses showed that the maximum temperature reached is around 62 °C, compared to a working temperature of 20 °C, with a maximum absolute deformation of 60 µm, resulting in negligible effects on the resonance frequency of the structure.

The results have led to the identification of a specific final cavity design, which will be built in the following months. The developed device demonstrated satisfactory performance along with considerable manageability in its realization, rendering the designed element suitable for high-power coupler testing for the RFQ of the Anthem Project.

## Figures and Tables

**Table 1:** Transmission line equivalent circuit and the HFSS simulation results comparison.

	TL Circuit	HFSS Simulation
l [mm]	425.6	429.8
Δf [kHz/mm]	833.3	820

**Table 2:** Coaxial resonator approximation and the HFSS simulation result comparison.

	Coaxial Resonator	HFSS Simulation
β	5.35	5.97
$P_{cav}\backslash P_{in}$ [%]	15.6	14.3

**Table 3:** Evaluated parameters for thermal design.

	Plate Channel	Longitudinal Channel	Coaxial Channel
Channel number	5	8	4
$D\left[mm\right]$	8	6	2
$m\left[\frac{l}{min}\right]$	6	3.5	4
$v\left[\frac{m}{s}\right]$	2	2	2
$h\left[\frac{W}{m^{2}{}^{\circ}C}\right]$	8600	9100	12,000
$r\left[\frac{bar}{m}\right]$	0.7	0.1	0.4

## Data Availability

The data are contained within the article.
